# Synthesis and Characterization of Polyaniline-Chitosan Patches with Enhanced Stability in Physiological Conditions

**DOI:** 10.3390/polym12122870

**Published:** 2020-11-30

**Authors:** Sami Ur Rahman, Salma Bilal, Anwar ul Haq Ali Shah

**Affiliations:** 1 National Centre of Excellence in Physical Chemistry, University of Peshawar, Peshawar 25120, Pakistan; samiurrahman364@yahoo.com; 2 Institute of Chemical Science, University of Peshawar, Peshawar 25120, Pakistan

**Keywords:** conductive polymeric patch, electroresponsive tissues, sodium phytate, aniline, chitosan, physiological medium

## Abstract

Electroconductive polymeric patches are being developed in the hope to interface with the electroresponsive tissues. For these constructs, conjugated polymers are considered as conductive components for their electroactive nature. Conversely, the clinical applications of these conductive polymeric patches are limited due to their short operational time, a decrease in their electroactivity occurs with the passage of time. This paper reports on the polymerization of aniline on prefabricated chitosan films on microscopic glass slides in the presence of sodium phytate. The strong chelation among sodium phytate, aniline and chitosan led to the formation of electoconductive polymeric patch. We assume that immobilization of sodium phytate in the polymeric patch helps to prevent electric deterioration, extend its electronic stability and reduce sheet resistance. The patch oxidized after three weeks (21 days) of incubation in phosphate buffer (pH 7.4 as physiological medium). This feasible fabrication technique set the foundation to design electronically stable, conjugated polymer-based patches, by providing a robust system of conduction that could be used with electroactive tissues such as cardiac muscles at the interface.

## 1. Introduction

Among the conductive materials used in bioelectronics, conducting polymers (CPs) have attracted much attention over recent years because of their ability to conduct both electronically and ionically [1], to be processed into electroconductive polymeric patches [2] and to be rendered biodegradable [3,4]. Electroconductive polymeric patches are a synthetic support for building a three dimensional (3-D) construct that is functionally, structurally and mechanically equal to or better than the tissue that is to be replaced or repaired [1,2]. In tissue engineering, bioelectronics devices based on these conductive polymeric patches are being developed with the anticipation of re-establishing communication between interrupted cells. Low-resistance pathways in excitable tissues, such as the heart, nerves and muscles, permit rapid cell-to-cell communication via currents generated by the flow of ions between neighboring cells [5]. These polymeric patches are critical for the proper function of excitable tissues. They play a very significant role in the regeneration of the damaged tissue by providing a structurally relevant environment [6]. Biologically applicable patches have (1) porous interconnected structure, (2) biocompatibility and biodegradability, (3) mechanical strength for specific application and (4) modified surface morphology to support cell attachment and growth.

Numerous polymeric patches with various ingredients have been synthesized for the regeneration of different tissues. Intrinsically conducting polymers (ICPs) have gained great attention for applications in the field of tissue engineering [7]. Considering the vast amount of new possibilities polyaniline (PAni) offers, we believe it will revolutionize the world of tissue engineering. Unfortunately, its use in biological applications is limited by its low processability, lack of flexibility and nonbiodegradability, and has been noted to cause chronic inflammation once implanted [8,9]. It has been reported that all the above-mentioned properties of PAni to biomolecules can be improved through copolymerization with natural biocompatible and biodegradable polymers having a variety of functional groups such as –OH, –COOH, –NH_2_ and acetyl group [10].

Among various natural biopolymers, chitosan is a suitable candidate because of its remarkable hydrophilicity, biocompatibility and antibacterial properties, having hydroxyl and amino groups in their back bone that can be easily altered. These properties in a polymer are essential for the use in biomedical applications [11,12]. Therefore, conductive composites have been prepared by incorporating PAni into chitosan, combining the good biocompatibility and biodegradability of the chitosan and the electrical conductivity of the PAni [13,14]. Chitosan-grafted PAni has found its application in various biological fields [15].

Shukla and Tiwari, 2011 [13], reported preparation of chitosan-grafted PAni through oxidative radical copolymerization at room temperature using CuSO_4_ as a polymerizing agent. The grafted composite material showed improved electrical conductivity (~10^−6^ S/cm) due to the PAni incorporated onto the chitosan. Furthermore, the composite was responsive to H^+^ ion, which is a suitable property of this composite for fabrication in biosensor devices. Sedaghat, 2014 [10], carried out chemical grafting of PAni on chitosan in the presence of H_2_SO_4_ and ammonium persulphate (APS) as a dopant and an oxidant, respectively. He reported that the synthesized material with relatively low conductivity with smooth and slick morphology can find applications in polymer-resistant coating, chemical sensors and storage containers. Moutsatsou et al., 2017 [16], fabricated a composite nanofibrous membrane containing PAni and chitosan by the electrospinning method at the evaluation of their biocompatibility. The membrane showed biocompatibility and supported cell growth and attachment without any toxic effects. The hydrophilicity retention and conductivity in the membrane was due to chitosan and PAni, respectively. Kushwaha et al., 2018 [17], synthesized chitosan-grafted PAni through in situ polymerization and suggested the homogeneous morphology and improved stability of grafted copolymer. They also deposited a film of grafted copolymer on indium tin oxide (ITO) substrate for urea sensing and found good sensing properties. The solution casting method was reported by Pasela et al., 2019 [18], to synthesize PAni-chitosan composites. They investigated that PAni did not affect the biocompatibility of chitosan and can be utilized for biomedical applications. Lee et al., 2019 [19], prepared a Chitosan-grafted PAni, to develop a sensitive device which responds to the presence of hazardous acids. The synthesized, chitosan-grafted PAni showed good solubility in common polar organic solvents and excellent film-forming properties.

Although in the field of tissue engineering, bioelectronics devices have been developed by using polymeric patches such as chitosan-grafted PAni with the expectation to restore communication among interrupted cells [3,4]. However still under physiological conditions, de-doping of PAni or other ICPs is a serious issue for its long-term application in medical and biological fields [20]. Therefore, further investigation is highly necessary to select appropriate dopants for ICPs that can maintain the conductive nature of these implanted materials in physiological medium.

In the present work, aniline was polymerized on a prefabricated film of chitosan using ammonium persulphate (APS) as an oxidant and sodium phytate as a novel dopant. Taking advantage of the cationic amine groups present on PAni and the chitosan backbone and the anionic (multivalent) nature of sodium phytate, the chitosan-PAni patch is expected to maintain its conductive nature by retaining the dopant. The insulating nature of the chitosan is advantageous for using as a substrate on account of its malleability, mechanically tough and detachable conductive film formation without affecting the electrical behavior of PAni. The use of facile fabrication method is expected to be helpful for the utilization of polymeric patches in tissue engineering.

## 2. Materials and Methods

### 2.1. Materials

Aniline (C_6_H_5_NH_2_), sodium phytate (C_6_H_17_NaO_24_P_6_), ammonium persulphate ((NH_4_)_2_S_2_O_8_ and chitosan flakes were obtained from Sigma Aldrich (St. Louis, MI, USA) and acetic acid was purchased from Arcos organics (4823 Newton Dr, Carlsbad, CA, USA). Fluorine-doped tin oxide (FTO) glass (13 Ω/sq) was obtained from Solaronix, Aubonne, Switzerland. All chemicals used in this research project were of analytical quality and used without further purification except Aniline. Aniline was freshly double-distilled to remove any type of impurities. After double distillation, the aniline was kept in the refrigerator for further use. All the samples were prepared on Pyrex glass wares. Deionized (DI) water was used in all this study for sample synthesis and washing purposes.

### 2.2. Preparation of Chitosan Viscous Solution

The chitosan flakes (1% (*w*/*v*)) (medium molecular weight 85% deacetylation) were dissolved in 1% (*v*/*v*) aqueous acetic acid solution. This solution was continuously stirred for three hours under room temperature to obtain a viscous solution of chitosan with pale-yellow color. After this, centrifugation of the viscous solution was carried out for 10 min at 6000 rpm for the removal of any insoluble flakes. After centrifugation, this solution was stored as a stock solution for the preparation of chitosan films on microscopic glass slides.

### 2.3. Fabrication of Chitosan Film on Microscopic Glass Slides

**Scheme 1:**
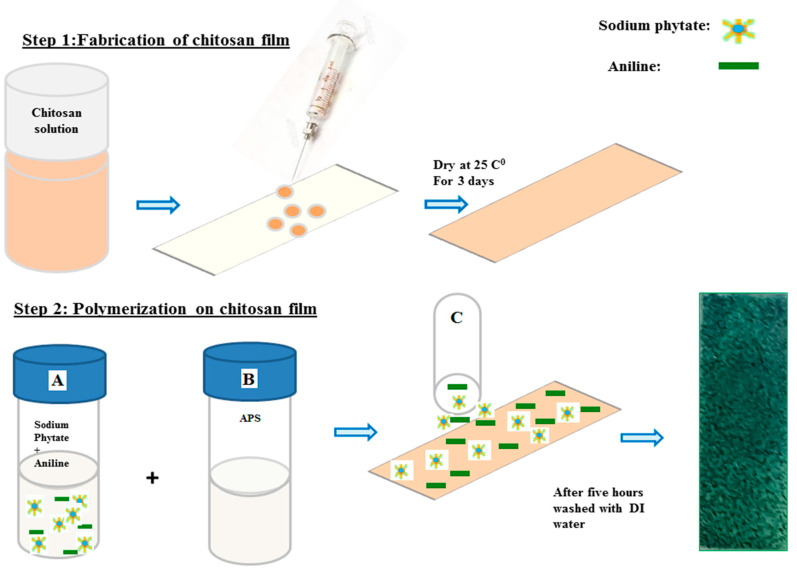
Step 1: fabrication of chitosan film on microscopic glass slide. Step 2: polymerization of aniline on the chitosan film. DI: deionized water; APS: ammonium persulphate.

Chitosan solution (0.75 mL) was uniformly spread on the surface of microscopic glass slides (7 cm × 2 cm) (Scheme 1, Step 1) and left to dry for three days at normal atmospheric pressure and room temperature.

### 2.4. Fabrication of Chitosan-PAni Conductive Polymeric Patches

For the fabrication of chitosan-PAni patches, different solutions with 0.5%, 1%, 3%, 5%, 7% and 10% (*w*/*v*) sodium phytate were prepared in DI H_2_O at room temperature. Then 2.5 mL from the respective sodium phytate solution was mixed with 497 μL aniline in 5 mL H_2_O (solution A). A second solution containing ammonium persulfate (1.0 mM in H_2_O) was prepared (solution B). These solutions were kept in a refrigerator for 15 min. After that, for the fabrication of chitosan-PAni conductive patches, 0.5 mL of solution A was added to 0.25 mL of solution B in an Eppendrof tube for each patch (solution C), followed by thorough mixing, and then uniformly dispersed on the surface of the prefabricated film of chitosan (Scheme 1, Step 2), and permitted to polymerize on the surface of the chitosan films for five hours. The change in color from light brown to dark green indicated the polymerization on the surface of chitosan film. After five hours, these patches were rinsed extensively with DI water to wash out the unreacted dopant, uncross-linked polymeric chain or any type of other side products formed during the polymerization. After drying, the patches were carefully peeled off from the surface of the microscopic glass slides and each patch was placed in between the two microscopic glass slides for the maintenance of its flat shape. The respective chitosan-PAni patches were labeled according their amount of dopant with patch-0.5 for the lowest to patch-10 with the highest dopant concentration.

### 2.5. Material Characterization

#### 2.5.1. In Vitro Characterization Physical Properties of the Patch

For surface morphology, all polymeric patches were analyzed by scanning electron microscopy (SEM) (Helios G4 CX Dual Beam microscope equipped with Octane Elite, Berlin Germany) within the voltage range of 5 kV. To transform the sample for SEM a suitable amount of each sample was placed on aluminum stubs by using conductive taps. For each sample an image was taken using a focused electron beam under suitable resolution and voltage. Elemental composition and mapping, was carried out by using Helios G4 CX FEI Deutschland GmbH, Berlin, Germany. Sheet resistance was checked by a Four Probe Conductometer (Jandel RM 3000, Jandel Engineering Ltd., Linslade, Bedfordshire, UK) equipped with a potentiostate. Atomic force microscopic (AFM) imaging was done through a 2000 nm × 2000 nm scan area via NanoWizard^®^ 3 Bio AFM JPK/Bruker, Berlin, Germany. The roughness of the patches was calculated from 2-D Height AFM images using image data processing software JPK Nanowizard. For functional groups conformation FT-IR Spectrometer (Shimadzu, Tokyo, Japan), was used, the spectra were recorded from 400–4000 cm^−1^.

#### 2.5.2. In Vitro Characterization/Electronic Properties of the Patch

Cyclic voltammetry measurements were recorded by using ZRA Potentiostat/Galvanostat Reference 3000 (Gamry Instruments, Warminster, PA, USA). The experiments were carried out by using gold coil and Ag/AgCl as counter and reference electrode, respectively. The patch was fabricated on the surface of FTO and was used as a working electrode. The electrochemical measurements were performed in the potential range of −0.2 to 0.8 at a scan rate of 30 mV s^−1^ in phosphate buffer solution having pH 7.4 as physiological medium. UV-vis experiments were performed on a LAMBDA 1050 from Perkin Elmer (Waltham, MA, USA). For UV measurements film with reduced opacity was incubated in a cuvette containing phosphate buffer solution and allowed for the transmission of the UV beam. The absorption spectra were recorded at a predetermined time in the range between 300–1000 nm.

## 3. Results and Discussion

### 3.1. Surface Morphology of the Patches Using SEM

**Figure 1:**
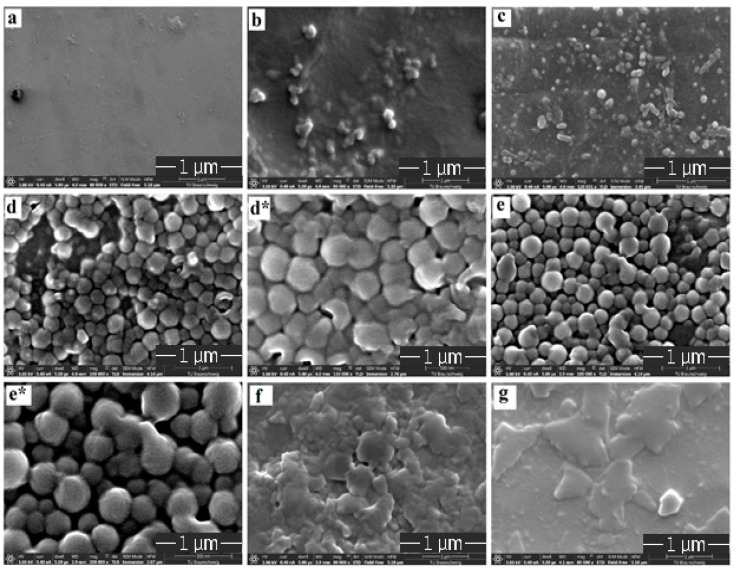
SEM images of (**a**) chitosan, (**b**) patch-0.5, (**c**) patch-1, (**d**) patch-3, (d*) zoomed image of patch-3, (**e**) patch-5, (e*) zoomed image of patch-5, (**f**) patch-7 and (**g**) patch-10.

Morphology of the pristine chitosan and chitosan-grafted-PAni patches were investigated by high-resolution SEM and are shown in Figure 1a–g. No visible pores were observed for the pristine chitosan patch (Figure 1a). On polymerizing the aniline on chitosan films, as the dopant (sodium phytate) concentration increases from 0.5% to 5% (*wt*/*v*) the surface gradually become rough with a uniform, granular nanostructure as for patch-3 and patch-5 (Figure 1d,e). With further increase in the dopant amount up to 10% (*wt*/*v*), the granular morphology completely disappeared due to agglomeration of the particles on the chitosan surface (Figure 1f,g).

From the SEM images of the patches, patch-3 and patch-5 present a homogeneous interlinked granular morphology. Comparative to smooth morphology of the chitosan film (substrate) as shown in Figure 1a, patch-3 and patch-5 have a rougher surface topography illustrating that the polymerization results in the uniform distribution of PAni on the surface of chitosan. The granular structure of PAni in the chemical polymerization is an important characteristic of PAni [21].

Comparison of patch-3 with patch-5 shows that granular particles are arranged compactly with each other having much less porosity while in the case of patch-5, uniform, granulated particles are arranged in an interlinked manner having pores between the granules. The homogenous interlinked granular porous morphology in patch-5 can be explained due to the existence of the fine proportion of both the monomers and the dopant. This optimization increases the electrostatic repulsion within the PANI chain and is responsible for the domination of the extended chain formation. Further increase in dopant concentration leads to the hydrolysis of the polymeric granular structure which results in the agglomeration and complete disappearance of the uniform, granular morphology. A uniform, granulated and porous structure has great importance in biological fields. Therefore, it can be expected that patch-5 has the most promising and desirable morphology amongst all the patches.

From these results, we can say that sodium phytate imparts a significant effect on the surface modification. Due to the anionic nature of sodium phytate, amine groups of chitosan can be protonated and bind to the PAni (positively charged) resulting in the formation of a blended system with strong interactions between its components [22,23]. The use of a facile fabrication technique for the utilization of PAni patches in terms of conductive polymeric patches is very significant because it provides a platform to affiliate with electroresponsive tissues like the heart [1].

### 3.2. Morphological Studies of Patch-5 Using Atomic Force Microscopy (AFM)

**Figure 2:**
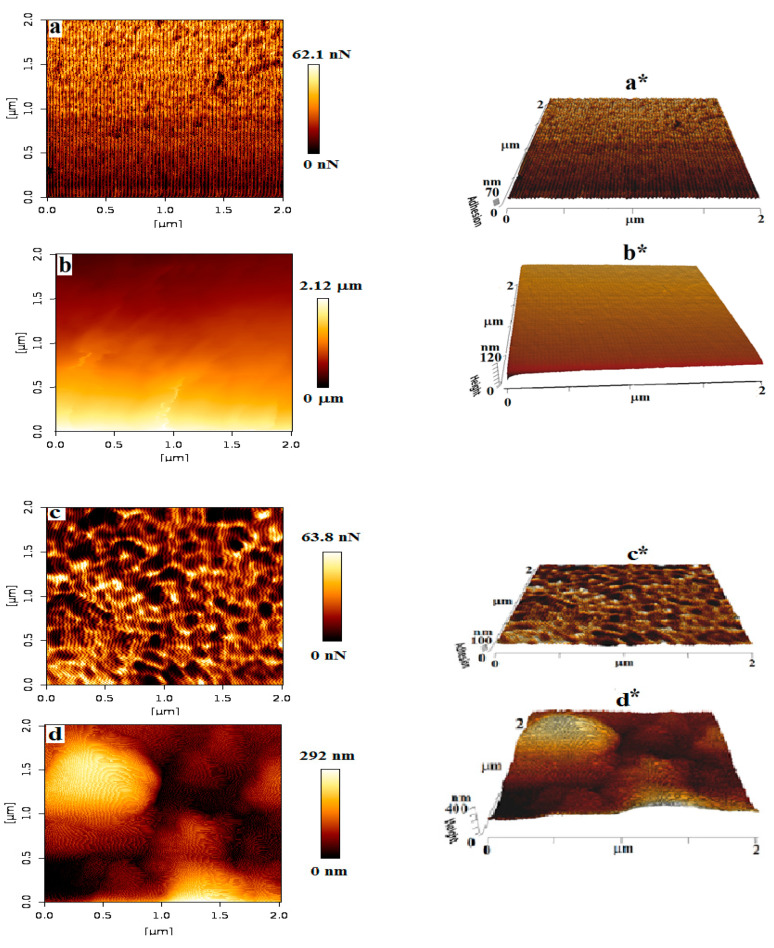
Atomic force microscopic (AFM) image of chitosan (**a**,a*) 2-D and 3-D image of adhesion and (**b**,b*) 2-D and 3-D image of height; and patch-5 (**c**,c*) 2-D and 3-D image of adhesion and (**d**,d*) 2-D and 3-D image of height.

The surface morphology of the pristine chitosan and the most optimized patch i.e., patch-5 was further confirmed with AFM, to gain insight into the uniform polymerization. For each, pristine chitosan patch and patch-5, 2-D (adhesion and height) and 3-D (adhesion and height) phase images were captured to explore the morphological characteristics of the patches before and after polymerization. Figure 2a–d displays the AFM images of the pristine chitosan and patch-5, respectively. From the AFM images it can be seen that before polymerization the surface of chitosan (Figure 2a) is smooth having no porosity, while after polymerization patch-5 presents a porous network composed of a homogeneously distributed, interconnected granular morphology as shown in Figure 2c. From the extracted profile of the 2-D images, roughness of the pristine chitosan film (Figure 2b) and patch-5 (Figure 2d) was measured (Table 1). From the roughness results it could be observed that patch-5 has a rough and well-defined granular structure as shown in Figure 2c, relative to the pristine chitosan film. The rough and porous topography indicates that polymerization of PAni have proceeded effectively and uniformly on the chitosan surface [24,25]. These results are consistent with the surface morphology observed through SEM for patch-5. This uniform, interconnected granular surface of the patch-5 is desirable in tissue engineering especially in the cardiac muscles [26].

### 3.3. Sheet Resistance of the Patch

The sheet resistance of the patch-5 was measured by four-probe method (Table 1). The patch-5 shows low sheet resistance across the surface. This low sheet resistance of the patch-5 corresponds to the uniform, nanoscale-interconnected granular network-like structure which is clear from SEM and AFM analysis. This uniform, interconnected granular structure of patch-5 reduces the inter-chain separation between the PAni backbones [26]. Therefore, patch-5 presents the optimal electrical property, roughness, porosity and interconnected granular morphology thus can be a potential candidate for tissue engineering especially in electrochemical-responsive tissues such as cardiac muscles as the above-mentioned properties are reported to be beneficial for repairing such types of tissues [21,27].

### 3.4. Energy-Dispersive X-ray Spectroscopy and Elemental Mapping

**Figure 3:**
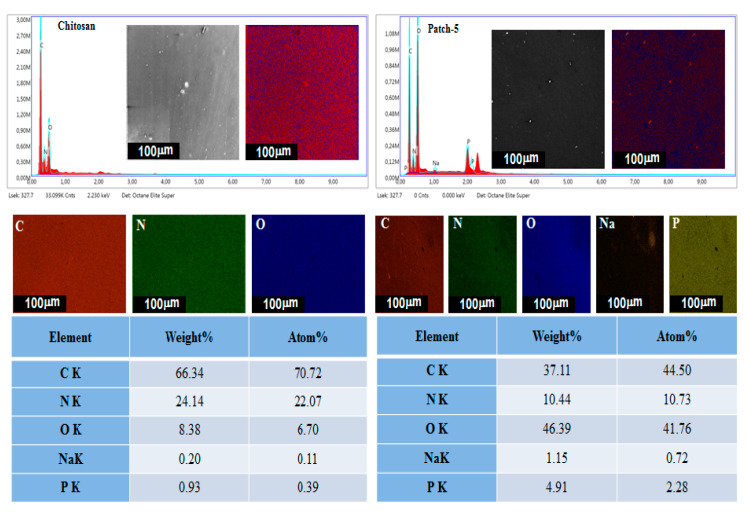
EDX and elemental mapping of pristine chitosan and patch-5.

To investigate the elemental composition of pristine chitosan and chitosan-PAni optimized polymeric patch-5, EDX analysis and surface mapping of elements was employed and the results are presented in Figure 3. The results of EDX spectra demonstrate that the composition of pristine chitosan consists of C, N and O with detected atomic ratios of 52.89%, 21.53% and 25.58%, while chitosan-PAni patch-5 is mainly composed of C, N, O, Na and P. The detection of P and Na peaks revealed the successful incorporation of sodium phytate as a dopant in to the polymer back bone, because the dopant molecules are responsible for the introduction of corresponding peaks in patch-5. The detected atomic ratios of P and Na are 2.28% and 0.72% for patch-5. The EDX elemental maps suggest the uniform distribution of P and Na along with other elements, leads to the uniform and cross-linked granular, network-like structure with low sheet resistance [28].

### 3.5. FTIR Spectroscopy

**Figure 4:**
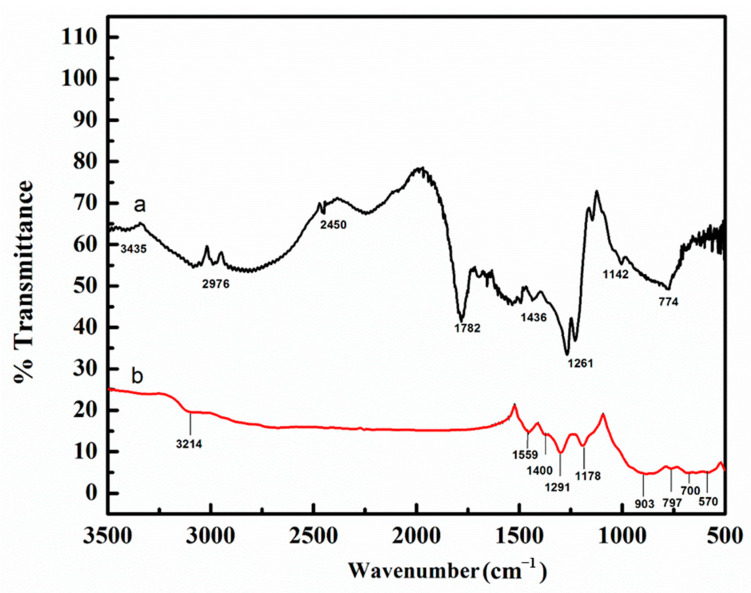
FTIR spectra of (**a**) chitosan film and (**b**) patch-5.

Figure 4a,b shows the comparison of FTIR spectra of chitosan and polymeric patch-5. The chitosan film spectrum (Figure 4a) showed an expected absorption peak at 3435 cm^−1^ (–NH_2_ stretching) and the bands 1782–1436 cm^−1^ (–NH_2_ bending). The band located at 2976 cm^−1^ is assigned to the C–H stretching mode in the chitosan [29,30]. The peak at 1261 cm^−1^ indicates asymmetric stretching of C–O–C bridge, while the peak at 1142 cm^−1^ corresponds to the skeletal vibration of the C–O stretching. These peaks are characteristic of its saccharide structure [31]. The patch-5 infrared spectrum (Figure 4b) manifests all the characteristic peaks corresponding to chitosan and PAni. The band observed at 3214 cm^−1^ depicts –NH_2_ stretching with 20 amino groups in chitosan [32]. The bands observed at 1559 cm^−1^ depict the stretching mode of C=O, it is generally due to saccharide [33]. The appearance of C=C stretching mode of quinoid ring and benzenoid ring at 1400 cm^−1^ and 1291 cm^−1^, respectively are characteristics of PAni [28]. The bands at 1178 cm^−1^ are attributed to the C–N stretching. The detection of bands at 903 cm^−1^ depicts the asymmetrical C–O and is generally due to the saccharide structure [34]. The appearances of the bands in the range of 797 cm^−1^ are allocated to vibration of P–O bond [35,36]. The band around 700 cm^−1^ is associated with P–O–C stretching vibration [37]. The bands at 570 cm^−1^ represent the O–Na stretching vibration [29]. Therefore, the detection of respective bands in the spectrum of patch-5 should be attributed to the incorporation of sodium phytate into the PAni back bone indicating the successful polymerization of aniline on the chitosan film.

### 3.6. Cyclic Voltammetry (CV)

**Figure 5:**
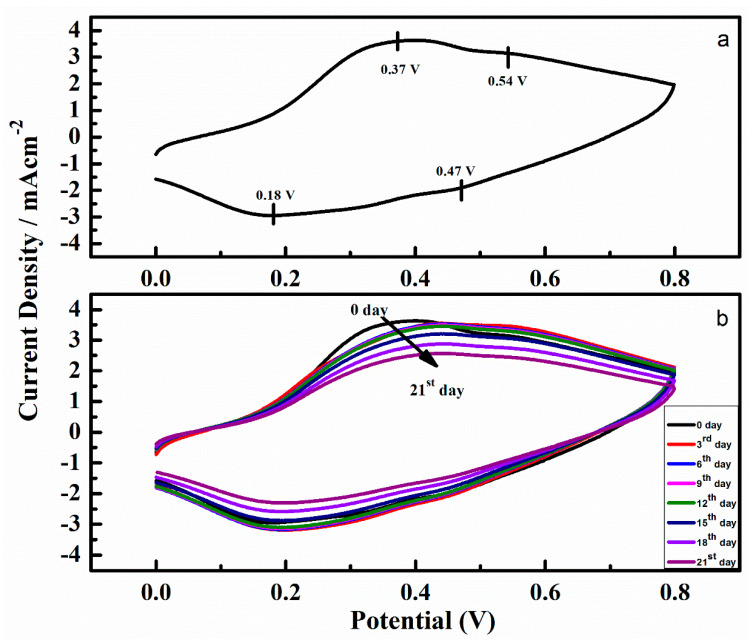
(**a**) Cyclic voltammogram (CV) of patch-5, (**b**) CV curves of patch-5 recorded at the 0th, 3rd, 6th, 9th, 12th, 15th, 18th and 21st day in phosphate buffer.

Figure 5a,b demonstrate CVs of the optimized patch-5 before and after incubation in phosphate buffer solution. From the Figure 5a, it can be noted that the obtained cyclic voltammogram have characteristic emeraldine salt features of PAni, indicated by two redox couples, a primary couple at 0.37 V (anodic) and 0.54 V (reduction) and a secondary couple at −0.18 and −0.47 V. Although the CV measurement was carried out in phosphate buffer with a pH 7.4, still these characteristics are identical to a typical PAni in a pH less than 6.0, this is due to the internal environment in chitosan-PAni patch produced by the dopant molecules. Further, we recorded the CVs after 3, 6, 9, 12, 15, 18 and 21 days of incubation in the phosphate buffer, displayed in Figure 5b. From the CV curves it can be noticed that gradually a slight increase in peaks separation occurred indicating that charge transfer becomes difficult with the passage of time. After 21 days, the only primary redox couple appears, as expected for more alkaline pH [38]. The patch remained conductive over the entire incubation period in a neutral pH, unlike the PAni films doped with other conventional acids that show negligible electrochemical properties at a pH less than 4, because the dopant is lost [39,40]. The electrochemical activity observed from the CV curves for the polymeric patch-5 in phosphate buffer solution illustrate that incorporation of the dopant sodium phytate during the polymerization of aniline on chitosan film is a feasible methodology to produce conductive materials that show electrocatalytic activity under a physiological-relevant environment for an extended period of time. While the patch included a substantial amount of the insulator chitosan film as a substrate, after polymerization of aniline, it shows that an effective amount of charge transport occurs through the polymeric patch even in the phosphate buffer as physiological medium.

### 3.7. UV-Vis Spectroscopy

**Figure 6:**
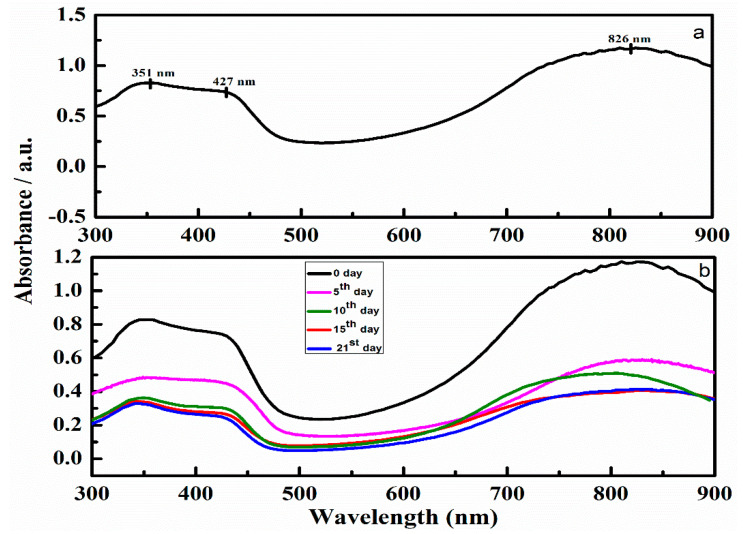
(**a**) UV-visible spectrum of patch-5 and (**b**) UV-visible spectra of patch-5 recorded at 0th, 5th, 10th, 15th and 21st day.

The electronic structure of PAni (Figure 6a) could be examined by observing the characteristic absorbance peaks at 351 nm due to p-p* transition of the benzenoid group, 427 nm due to polaronic shoulder and 826 nm due to the polaron region. The peak at 427 nm corresponds to the electrically conductive state of PAni, the emeraldine salt [38]. Beside this, another broad peak can also be noticed in the region between 793 and 826 nm, which is also another typical peak for the conductive emeraldine salt form of PAni [41]. To understand the effect of the incubation time on the electronic structure of the synthesized polymeric patch, a sample of the patch-5 was incubated in phosphate buffer solution and the UV-vis spectra were recorded at different time intervals as shown in Figure 6b. To evaluate the effect of incubation time on the protonated form of the polymeric patch, we concentrated on the peak in the range of 422–427 nm. From Figure 6b it can be observed, as the incubation time increases, a decrease in the wavelength along with absorption of the peak from 427 to 422 nm occurs, suggesting that the level of doping decreases with the incubation time of the patch in the phosphate buffer solution. This decrease in the peak was attended by a blue shift in the region of polaron centered at 793 nm indicating a decrease in the conductive state of the patch. The shifting of the polaronic peak from 826 to 793 nm suggests that the fabricated patch is still in conductive form even after 21 days of incubation period. These changes observed in the spectral peaks agree with the changes in CV curves (Figure 5b). Thus the results obtained from the UV demonstrate that the patch maintained protonated species for 3 weeks in phosphate buffer solution, because of the peak presence in the region of 427 to 422 nm in all measured spectra.

These results indicate that our methodology of polymerizing the aniline in the presence of sodium phytate as a dopant leads to an electroconductive polymeric patch of PAni that shows conductivity for an extended period of time in physiological conditions.

## 4. Conclusions

Polymerization of aniline was carried out using sodium phytate as a dopant and APS as an oxidant on chitosan film prefabricated on microscopic glass slides to obtain electroconductive polymeric patches. According to the concentration of the dopant, six polymeric patches i.e., patch-0.5, patch-1, patch-3, patch-5, patch-7 and patch-10 were synthesized. From SEM results strong chelation between the dopant, chitosan and aniline was observed in the case of patch-5 which showed interconnected uniform nanoscale granular morphology. The surface topography of the optimized case patch-5 was further confirmed by AFM. Sheet resistance of the patch was measured by four-probe method and showed very low sheet resistance. Elemental composition and functional group detection was carried out by EDX and FTIR revealed the quantitative and qualitative characteristics of the fabricated patches. The results obtained from both the CV and UV show that the patch remains conductive for 21 days after incubation in a neutral conditions, utilizing phosphate buffer solution having pH 7.4, as a physiological medium. A uniform, nanoscale-interconnected granular surface with high electrocatalytic activity provide a promising electronic stability to patch-5 in physiological conditions that is desirable in tissue engineering, especially in cardiac muscles. Therefore, our feasible fabrication technique has set the foundation to design electronically stable PAni-based patches which will be possibly applicable in tissue engineering.

## Figures and Tables

**Table 1:** Roughness and sheet resistance of chitosan-grafted PAni patch calculated from 2-D, image of height and four-probe technique, respectively.

Sample Code	Roughness		Sheet Resistance (Ohm/cm^2^)
	Average Resistance (Ra)	Root Mean Square Resistance (Rq)	
**Pristine chitosan**	13.52 nm	20.19 nm	-
**Patch-5**	51.86 nm	66.31 nm	14.15

## Dedication

This paper is dedicated to Rudolf Holze, Chemnitz University of Technology, Germany, on the occasion of his 65th birthday.
